# HIV Education and Welfare Services in Primary Care: An Empirical Model of Integration in Brazil’s Unified Health System

**DOI:** 10.3390/ijerph14030294

**Published:** 2017-03-14

**Authors:** Rahbel Rahman, Rogério M. Pinto, Melanie M. Wall

**Affiliations:** 1 Department of Social Work, Community of College and Public Affairs, Binghamton University, 67 Washington St, Binghamton, NY 13902, USA; 2 School of Social Work, University of Michigan, 1080 S University Ave, Ann Arbor, MI 48109, USA; ropinto@umich.edu; 3 Department of Biostatistics, Columbia University, 722 West 168th St. New York, NY 10032, USA; mmw2177@cumc.columbia.edu

**Keywords:** HIV/AIDS, social services, service integration, interprofessional collaboration

## Abstract

Integration of health education and welfare services in primary care systems is a key strategy to solve the multiple determinants of chronic diseases, such as Human Immunodeficiency Virus Infection and Acquired Immune Deficiency Syndrome (HIV/AIDS). However, there is a scarcity of conceptual models from which to build integration strategies. We provide a model based on cross-sectional data from 168 Community Health Agents, 62 nurses, and 32 physicians in two municipalities in Brazil’s Unified Health System (UHS). The outcome, service integration, comprised HIV education, community activities (e.g., health walks and workshops), and documentation services (e.g., obtainment of working papers and birth certificates). Predictors included individual factors (provider confidence, knowledge/skills, perseverance, efficacy); job characteristics (interprofessional collaboration, work-autonomy, decision-making autonomy, skill variety); and organizational factors (work conditions and work resources). Structural equation modeling was used to identify factors associated with service integration. Knowledge and skills, skill variety, confidence, and perseverance predicted greater integration of HIV education alongside community activities and documentation services. Job characteristics and organizational factors did not predict integration. Our study offers an explanatory model that can be adapted to examine other variables that may influence integration of different services in global primary healthcare systems. Findings suggest that practitioner trainings to improve integration should focus on cognitive constructs—confidence, perseverance, knowledge, and skills.

## 1. Introduction

Across the globe, Human Immunodeficiency Virus Infection and Acquired Immune Deficiency Syndrome (HIV/AIDS) continues to disproportionately burden low-income ethnic/racial groups and sexual minorities who face myriad other chronic diseases [1,2,3]. In 2015, 830,000 people were living with HIV in Brazil, the prevalence for adults aged 15 to 49 was 0.6%, with the highest rates in populations facing low educational attainment and economic inequality [4]. HIV risk behaviors arise within the context of socioeconomic determinants of health, such as the physical, familial, cultural, organizational, economic, policy/legal, and social environments in which those affected live [5,6]. Given the scope of these determinants of health, governments worldwide, including Brazil, have developed workforces to provide HIV/AIDS prevention education while also assisting low-income individuals to obtain documentation (e.g., birth certificates, working papers, identity cards, etc.) for receiving primary care and welfare services (e.g., provision of nutritious food, etc.) [7,8].

Lack of consensus on a definition for service integration (e.g., in this case, the integration of HIV education with community activities and documentation services) has delayed research on the inclusion of different services into primary care [9,10]. “Service integration” by various authors has been used to refer to integrated care, continuity of care, coordinated care, managed care, comprehensive care, and patient-centered care [11]. There have been a number of studies that have confirmed the feasibility of integrating different services with primary care, such as mental health and drug treatment [12,13]. Integration has been shown to be a cost-effective tool [14] with the potential to improve disease treatment response, increase the likelihood of remission [15,16] and access to services [17], and improve patient well-being [17]. What remains unknown is how best to integrate HIV education with welfare-related services within primary care systems [10].

This paper advances the literature by providing an explanatory framework within which to build further research on service integration. We define “service integration” as the coordinated behaviors of practitioners in the Estratégia Saúde da Família (FHS; Family Health Strategy), the primary care program of Brazil’s Sistema Único de Saúde (UHS; Unified Health System), which offers multiple services to individuals at risk for multiple disorders and diseases. The UHS employs physicians, nurses, and Agentes Comunitários da Saúde in Portuguese or in English as community health agents (CHAs), all charged with the integration of welfare and public health services within primary-care units across the country [18]. The purpose of our study was to identify significant predictors of service integration at the practitioner and organizational levels along with practitioners’ job characteristics. The authors believe that the framework can be adopted by other researchers to study the integration of welfare services with other chronic diseases, such as tuberculosis (TB), cancer, etc.

### 1.1. Service Integration in Brazil’s Family Health Strategy

The FHS has institutionalized the provision of health and welfare services to comply with Brazil’s 1988 federal constitution and the 1990 Lei Orgânica da Saúde (Organic Health Law) [19]. The FHS offers free primary health care through a decentralized system, deploying interprofessional teams, each comprised of one physician, a nurse, two or three nurse assistants, and four to six CHAs. Each team provides care to between 800 and 1000 families, representing some 4000 individuals [20]. Collectively, the FHS teams provide primary care to more than 60 million Brazilian citizens [19]. According to Brazil’s National Standards, physicians, nurses, and CHAs, are involved in the provision of services in three key domains, as follows. HIV education comprises teaching community residents about how to access HIV testing and free condoms, how to use condoms, and how to practice safe needle exchange [21]. The FHS is involved in helping patients obtain documentation, such as birth certificates, working papers, identity cards, etc., needed for receiving primary care and welfare services. One must be a Brazilian citizen to qualify for services, for example, the *cesta básica* (food basket), a program that offers nutritious food for families living below the poverty line. Documentation is a crucial issue in a universal healthcare system to track Brazil’s most prevalent health problems and prioritize interventions to address them [20,22]. FHS teams also involve residents in sociocultural activities, including education campaigns to improve HIV knowledge, address stigma, and debunk HIV myths [23].

### 1.2. Factors That Influence Integration

Knowledge base, skills, perseverance, confidence, perception of team efficacy, and familiarity with the communities all influence how practitioners integrate different services. Whereas nurses and physicians possess biomedical knowledge (i.e., etiology and epidemiology of diseases) [24], CHAs (or Community Health Workers (CHWs), as known globally) are trained in basic medical practices and are specifically hired to use their lived experiential knowledge to impart health-promoting behaviors [25]. CHAs may acquire their lived experiential knowledge through having greater familiarity with communities’ lifestyles, traditions, and habits than physicians and nurses are intended to have [26]. Physicians and nurses often lack training in building partnerships with consumers to stimulate change within local communities [22,27]. Nonetheless, CHAs may assist medical practitioners with socio-emotional counseling skills [25]. The process of sharing their skills and knowledge can enhance practitioners’ confidence to provide both social and medical services [28].

Work conditions and available resources, such as office space, medical supplies, and data management systems, all comprise organizational factors that influence how different practitioners in a health team contribute to integrating services [29,30]. FHS uses discrete, formalized assignments for staff, while encouraging all practitioners to work together to combine different services. Though the roles and responsibilities of each FHS practitioner might be specific, FHS practitioners are not precluded from providing those services. While physicians are charged with developing care plans, nurses perform nursing care, request laboratory tests, dispense medications, supervise CHAs, and engage in health promotion activities [31]. CHAs collect and manage household data on births, deaths, disease incidence, and immunization status of children—in addition to offering health promotion activities [32,33]. Therefore, FHS practitioners integrate services by pooling their knowledge, skills, and cognitive competencies discussed above [34].

Several job-related variables, practitioners’ assignments and their discretion to perform such assignments, may influence their decisions concerning when and how to integrate different services [35,36]. For example, federal guidelines encourage FHS practitioners to account for the input of consumers as co-creators in their own health care [37,38]. Interprofessional collaboration, another important influence in integration, is characterized by practitioners working side by side, applying diverse knowledge/solutions to health issues. Other job-related factors include “practitioner autonomy” and “skill variety” [35]. Autonomy gives practitioners confidence that they are able to deal immediately with a consumer’s health issues [39]. Skill variety is a concept that arises from practitioner autonomy; in order for practitioners to offer consumers a wider range of services, they must possess diverse skill sets, such as the ability to integrate health and welfare services into their practices [40].

CHAs are hired from the communities in which they live, but most nurses and physicians reside outside the poor communities that they serve [20]. Yet the practitioners’ socio-geographic relationships to the communities they serve has received little attention in the literature. In this study, we examined whether or not practitioners living closer to the communities they served were better able to tailor prevention services to the needs of those communities.

### 1.3. Theoretical Framework

**Figure 1:**
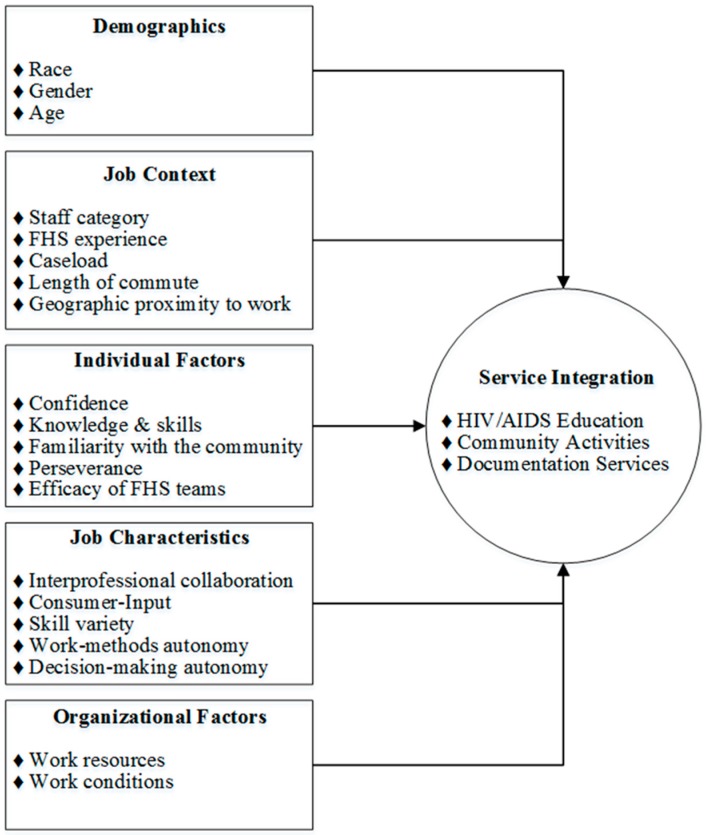
Service Integration Framework. FHS: Estratégia Saúde da Família (Family Health Strategy); Human Immunodeficiency Virus Infection and Acquired Immune Deficiency Syndrome (HIV/AIDS).

Our theoretical framework (Figure 1) reflects concepts of Cognitive-Behavioral Theory, Job Characteristics Theory [41], Modern Organizational Development Theory [42], and Structural Contingency Theory [43,44]. Cognitive Behavioral Theory reflects intrapersonal factors that influence practitioners’ service integration. Job Characteristics Theory suggests that practitioners’ integration of services can be influenced by their prescribed roles, by expected organizational norms, and by the presence or absence of organizational structure and of human and material resources. Practitioners are also influenced by their organization’s size/capacities, climate, and culture. Organizational theories, such as modern organizational developmental theory and structural contingency theory, explain how practitioners behave within community-based organizations, such as the FHS, that are systematized around provision of medical services.

## 2. Materials and Methods

This study was approved by the Institutional Review Boards of Columbia University, New York, NY, USA, (IRB-AAAC4674 (Y2M01)) and Universidade Católica, Rio de Janeiro, RJ, Brazil. This study followed a community-engaged strategy that included university partners, health care administrators, and CHAs who developed the study’s aims and methods and plans for disseminating findings [45]. Written consent was sought from all study participants. Collaborating with FHS administrators helped us to establish study aims that would produce results that would be beneficial to the communities in which the study would take place [46].

### 2.1. Sampling and Recruitment

We enrolled 168 CHAs, 62 nurses, and 32 physicians from 30 Unidades Básicas de Saúde (UBS), also known as community-based primary health care clinics, in two Brazilian municipalities. Through the UBS, FHS teams provide primary health care services. Each UBS had at least one physician (range = 1–2), one nurse (range = 1–5), and one CHA (range = 1–23). The average length of employment was 40 months (SD = 31; range = 4–156). Participation was voluntary. Brazil’s policy on research does not permit financial incentives; however, refreshments were provided during data collection.

### 2.2. Data Collection

Eight master’s level Brazilian interviewers were trained in research methods and procedures, and administered the survey using password-protected mobile computers. Data were downloaded into a password-protected database, DatStat Illume 4.6 (DatStat, Seattle, WA, USA) [47]. All data were kept in password-secured computer files, to which only relevant research personnel had access. There was no documentation linking respondents assigned ID numbers to the UBS for which they worked. The survey was administered verbally and lasted from 45 to 75 min. Approximately 85% of staff from all clinics participated.

### 2.3. Multidimensional Survey

Survey questions addressed participants’ perceptions of and attitudes toward their knowledge, skills and confidence, and job characteristics. We piloted the survey with 42 practitioners. CHAs showed difficulty understanding questions that tapped opinions and attitudes toward scientific research, and physicians found the survey too long. We used this input to modify the survey. Survey questions were then translated from Portuguese to English and iteratively back-translated into Portuguese [48] for accuracy.

### 2.4. Measures

All predictor variables appear in Table 1. The outcome, service integration, was measured by combining three dichotomous (yes/no) variables: (1) “I teach consumers how to prevent HIV and AIDS”; (2) “I help my consumers obtain documents, such as voter registration, working papers, and birth certificates”; and (3) “I help my consumers get involved in community activities, such as health walks and workshops.” Though each of the three integration variables may align more closely with the roles and job descriptions of one or another FHS provider, these services can be provided by all providers. Moreover, our measure of “integration” follows our conceptual framework in that integration is conceptualized as the combination of different services performed by different providers.

Demographics included “age” measured as a continuous variable; “gender” included male or female; “race” included Black, White, and *Pardo* (*Pardo* refers to mixed races, such as mulattos [49]).

### 2.5. Data Analysis

We summarized descriptive frequencies of demographics and job context variables. We investigated influences on FHS teams’ provision of HIV/AIDS education along with their involvement of consumers in community-level activities and helping them to obtain documentation/registration, “service integration,” as a latent variable underlying three measures: HIV/AIDS education; community activities; and documentation services (Figure 1). We used MPlus 7.1 software (Muthén & Muthén, Los Angeles, CA, USA) to fit the structural equation model following the form on Figure 1 using the weighted least squares estimation appropriate for categorical outcomes. The χ^2^ goodness of fit test, the ratio of the χ^2^ to the degrees of freedom, and the root mean square error of approximation (RMSEA) were used to assess fit. An RMSEA value of ≤0.05 signifies a good fit [51], and as the χ^2^ statistic tends to be over-sensitive to minor misfit, the ratio of the χ^2^ to the d.f (degrees of freedom) <2 is often considered good fit [52]. The percent of variability in the latent service integration variable explained by all the predictors in the model was used to quantify explanatory power of the model. Modification indices (MIs) were tested to examine any possible direct effects between the predictors and the three observed measures of service integration (above and beyond the effects through the latent service integration variable).

## 3. Results

### 3.1. Sample Characteristics

Table 2 shows that most respondents were CHAs (*n* = 169; 64%); 62 were nurses (24%); and 31 were physicians (12%). The highest proportion of respondents identified as Pardo (*n* = 123; 47%); 82 were White (32%); and 54 (21%) were Black. The majority were female (*n* = 214; 82%). Average age was 33.60 (SD = 9.99, range = 20–70). The majority of practitioners (*n* = 175; 67%) reported one to five years of working with FHS. 73% of CHAs and 60% of nurses had 1–5 years of experience. 46% of physicians reported spending 1–5 years with the FHS. The highest proportion of practitioners reported that they had ≤250 cases per month (*n* = 133; 51%); 91 (35%) reported their caseload was >501 cases per month, and 38 practitioners stated that their caseload was between 251 and 500 cases per month (14%). Seventy-seven percent of CHAs had ≤250 cases per month; 95% of nurses and 94% of physicians reported having >501 cases per month. Half of the practitioners reported that their commute to work was 0–10 min (*n* = 129; 50%); 88 (34%) stated their commute ranged from 11–30 min; and 43 (16%) over 30 min. Sixty-seven percent of CHAs reported that their length of commute was 0–10 min, 48% of nurses reported commutes of 11–30 min, and 55% of physicians reported commutes over 30 min. Ninety-one percent of CHAs said “yes” to the questions asking if they lived in proximity to their work; however, 55% of nurses and 84% of physicians answered “no”.

### 3.2. Predicting Service Integration

Of the 262 practitioners, 217 (83%) reported offering HIV-prevention services; 212 (81%) engaged in community mobilization; and 118 (41%) engaged in documentation services. Of the 169 CHAs, 142 (83%) reported offering HIV-prevention services; 138 (82%) reported mobilizing communities; and 85 (51%) stated they did documentation services. Fifty-three of 62 nurses reported offering HIV-prevention services (85%); 51 (81%) stated they mobilized communities; and 14 (23%) engaged in documentation services. Of 31 physicians, 22 (71%) offered HIV prevention services; 24 (77%) mobilized communities; and 8 (26%) offered documentation services.

Table 3 shows significant predictors of service integration. None of the demographic or job context variables were significant predictors of service integration, but practitioners with more experience trended toward higher service integration (B = 0.258; *p* = 0.060), and CHAs appear to be more involved in helping consumers obtain documentation/registrations.

Among the individual level factors, confidence (B = 0.322; *p* = 0.020), knowledge and skills (B = 0.448; *p* = 0.006), and perseverance (B = 0.237; *p* = 0.036) had a significant positive effect on integration. Among job characteristics, skill variety had a positive and significant association with service integration (B = 0.355; *p* = 0.017). Work-methods autonomy (B = −0.222; *p* = 0.097) and decision-making autonomy (B = −0.237; *p* = 0.075) trended toward a decreased association with service integration. Organizational factors did not show significant associations with service integration. Perseverance was a strong predictor (MI = 9.345) of the provision of HIV-prevention services, even above its effect on overall service integration. 

The summary model fit indices for the structural equation model were χ^2^ = 35.9 with degrees of freedom = 46, and the RMSEA was 0.001, 90% Confidence Interval (CI) (0.000–0.024). An RMSEA value of ≤0.05 signifies a good fit. The factor loadings of the three measures of service integration were all significant: HIV-prevention services –0.46, civil registration –0.43, community mobilization –0.48. Overall, the predictors in the model explained 62% of the variability in service integration.

## 4. Discussion

Integrating HIV prevention while helping consumers to obtain documentation and participate in community activities appears to be commonplace among FHS teams. Results show that greater FHS experience may predict better integration because practitioners who maintain long-term relationships with FHS consumers are likely to know their needs well and thus integrate the most needed services. The longer the time of their employment in the FHS, the better practitioners will be able to identify individual consumer behavior patterns that might lead to disease transmission, and the faster they will act to curtail those behaviors. CHAs are closest to the consumers they serve geographically and have greater knowledge of their community’s lifestyles, traditions, and culture than do other members of the FHS team. In making household visits, CHAs study their patients vis-à-vis their sociocultural practices [53]. In doing so, CHAs are the ones best able to identify individual HIV risk behaviors. They appear to be consistently engaging residents in community activities to learn HIV prevention [26,54].

Presumably, FHS team members’ awareness of one another’s expertise encourages them to consult one another about tasks or services needed for consumers and communities; this generates new knowledge for them. Such consultations ensure that services are integrated in accordance with consumers’ needs, and this, in turn, boosts consumer confidence. Service integration requires practitioners to use a combination of clinical, interpersonal, and advocacy skills, along with empathy and compassion toward consumers [18]. It has been established that physicians are given more autonomy than nonmedical practitioners, such as CHAs, [55], due to the perception that their medical and scientific knowledge is superior to the experiential knowledge of CHAs. Indeed, in Brazil, CHAs have reported having less autonomy in making decisions on how to integrate services [56]; nonetheless, our findings suggest that perhaps when motivated and with a sense of social closeness to the communities they serve, CHAs are able and willing to integrate services.

Our results showed that no organizational factors predicted service integration. This may be due to practitioners’ perception in recognizing Brazil’s poorly managed health system. Their exposure on a daily basis to corruption, lack of governance, and internal bureaucracy, all of which delays the process of buying and delivering supplies and medications, could result in practitioners perseverance in dealing with limitations of a universal health system and in seeking support of FHS team members while delivering integrated services to consumers [57]. However, it is important to note that these results stem from a secondary analysis of FHS data; our choices of quantitative measures were thus limited. Other organizational variables, such as practitioners’ job security, remuneration, and job satisfaction, may have improved the explanatory power of our model; however, it is also important to note that no realistic empirical model or dataset could contain every variable that might influence service integration.

Recall and information bias may have affected the reliability of this study’s findings. Practitioners who participated were asked to offer information about the previous six months; however, their attitudes might have changed during that time. Data were cross-sectional, making it impossible to determine causal associations; a longitudinal design would allow for a more comprehensive understanding of the associations between variables of interest. Our findings are not generalizable beyond the two municipalities where we collected data. Terms such as “HIV prevention services,” “work conditions,” and “work resources” were open to interpretation by respondents. The authors also identify that certain survey items, such as “I am able to make treatment plans which fit the needs and abilities of my patient”, may be double barreled and hence may have resulted in practitioners agreeing to the statement. We thus recommend further research to assess the best ways for measuring these variables, rephrasing survey items to avoid response bias, and include other variables that we did not examine. The authors also recognize that the survey instrument excluded questions on practitioners’ training and way of working through the stigma and discrimination faced by people living with HIV, especially those who are members of key populations such as Men who have sex with men (MSM), sex workers, etc. Variables that may improve the model include meeting minutes, assessments, reports, policies, inventory lists of medical equipment, and ways on how practitioners deal with the socio-structural factors impacting consumers. Job-related variables may include practitioners’ job security, remuneration, and job satisfaction. Future research may also include a larger sample, including FHS teams from multiple municipalities. Finally, we recommend longitudinal studies to examine relationships between the multi-level factors facilitating service integration and its effect on consumer outcomes. This will assist in creating sophisticated, evidence-based guidelines for service integration.

### Implications for Policy and Practice

Our explanatory model underscores key variables that might inform training to help physicians, nurses, and CHAs improve service integration. Practitioner trainings should focus on harnessing cognitive constructs, such as confidence, perseverance, and knowledge and skills. We recommend training that espouses active, problem-based, and action learning in order to reflect real-world practices. The need to address complex health promotion in the context of community factors calls for recognizing the limits of individual practitioner expertise so that practitioners can leverage the expertise of colleagues to provide comprehensive care to consumers. Diversity of expertise within FHS teams is important so that clinical expertise (physicians and nurses) may merge with experiential knowledge (CHAs) to solve complex health issues. We recommend that FHS health care managers/administrators work together to provide curricula/trainings that focus on service integration. Academic and FHS collaboration curricula ought to include the input of both CHAs and medical staff. The purpose of including CHAs in curricular design is to ensure that community-based skills are included in training/education initiatives.

## 5. Conclusions

This study shows that practitioners in Brazil’s FHS are likely to provide HIV education while helping individuals obtain documentation for receiving primary care and welfare services, and to participate in health promotion community activities. Our major contribution is an explanatory model that can be adapted to examine the impact of other variables that might influence integration of other services. This model can also be adapted to study service integration in primary care systems in other countries.

## Figures and Tables

**Table 1:** Predictors of Service Integration.

Predictors	Definition	Item(s) and Scale
**Individual Factors**		
Knowledge and skills	10-item composite: abilities to ask client/patients questions to provoke discussions about health, disease prevention, side effects of medications, and available resources (Cronbach α = 0.757)	5-point Likert scale (1 = strongly disagree to 5 = strongly agree) I know how to ask questions to help client/patients discuss their health I know how to ask questions about health risks I know how to ask questions about medication side effects
Confidence	3-item composite: appraisals of ability to provide client/patients with adequate services (Cronbach α = 0.521)	5-point Likert scale (1 = strongly disagree to 5 = strongly agree) I know exactly what my client/patient needs are I am able to make treatment plans which fit the needs and abilities of my patient I am able to address client/patient needs
Perseverance	Single item: extent of practitioner commitment to providing the best services	5-point Likert scale (1 = strongly disagree to 5 = strongly agree) I am committed to delivering the best services possible to the families in my catchment area, even when they are difficult
Efficacy of FHS teams	Single item: practitioner perception of team efficacy	5-point Likert scale (1 = strongly disagree to 5 = strongly agree) The existence of FHS teams has improved the quality of health in my catchment area
Familiarity with the community	Single item: extent to which practitioner knew the community	Dichotomous (yes or no) I know the latest news in my catchment area affecting client/patients
**Job Characteristics**		
Inter-professional collaboration	5-item composite: extent to collaboration between different professions (Cronbach α = 0.640)	5-point Likert scale (1 = strongly disagree to 5 = strongly agree) I utilize other colleagues in deciding interventions I have access to colleagues when I need help Team meetings are important
Consumer-Input	4-item composite: extent practitioner value/use client/patient input (Cronbach α = 0.627)	5-point Likert scale (1 = strongly disagree to 5 = strongly agree) My client/patient values and preferences are very important My client/patient goals are very important My client/patient and I work together to address needs With client/patients’ help, I monitor client/patient outcomes
Skill variety	3-item composite: extent of variety in skill sets (Cronbach α = 0.620)	5-point Likert scale (1 = strongly disagree to 5 = strongly agree) I am able to understand and use protocols I have the knowledge/skills to bring together information from different sources to address my client/patient’s needs I know how to use new information to treat my client/patient
Work autonomy	Single item: perception of work-related autonomy	Dichotomous: (0 = Disagree; 1 = Agree) I can tailor my work based on the information I gathered from my client/patient and from research
Decision-making autonomy	Single item: perception of work-decision-making	Dichotomous: (0 = Disagree; 1 = Agree) I am able to change or alter treatment based on changes in the needs of the client/patient
**Job context**		
Caseload	Single item: number of patients served	Continuous How many client/patients do you serve?
FHS Experience	Single item: years in the job	Continuous Tell us in years the length of time you worked for the FHS
Geographic proximity to work	Single item: perception of closeness to community	Dichotomous Do you live near your job? (yes or no)
Length of commute	Single item: minutes it takes to arrive to work from home	Continuous Tell us your length of commute (0–10 min; 11–30 min; and greater than 30 min).
**Work Environment**		
Work conditions	Single item: perception of quality of work conditions	Dichotomous (yes or no) Poor work conditions interfere with my ability to address needs of client/patient
Work resources	Single item: perception of quality of work resources	Lack of resources interfere with my ability to address needs of client/patient

All Cronbach alphas greater than 0.5, considered “reasonably good” when the subject matter under examination is novel (newly developed measures used in the analysis) ([50], p. 70); FHS: Family Health Strategy.

**Table 2:** Demographics and Job Context (Practitioner type).

Demographic Variables	CHAs	Nurses	Physicians	Total Sample	*p-*Value
	*n* = 169 (%)	*n* = 62 (%)	*n* = 31 (%)	*n* = 262 (%)	
**Race**					0.002 ^a^
Black	40 (24)	13 (21)	1 (3)	54 (21)	
White	42 (25.1)	22 (35)	18 (58)	82 (31)	
*Pardo*	85 (50.9)	27 (44)	11 (35)	123 (47)	
**Gender**					<0.001 ^a^
Male	28 (17)	4 (6)	16 (52)	48 (18)	
Female	141 (83)	58 (94)	15 (48)	214 (82)	
**Age**					0.138
20–30 years	76 (45)	27 (44)	12 (39)	114 (44)	
30–40 years	56 (33)	15 (24)	10 (32)	81 (31)	
41–50 years	27 (16)	12 (19)	3 (10)	42 (16)	
51–70 years	7 (4)	7 (11)	5 (16)	19 (7)	
**FHS experience**					0.008 ^a^
≤1 year	27 (16)	8 (13)	8 (26)	43 (16)	
1–5 years	123 (73)	37 (60)	15 (48)	175 (67)	
6–15 years	19 (11)	17 (27)	8 (26)	44 (17)	
**Caseload per Month**					<0.001 ^a^
≤250	131 (77)	2 (3)	-	135 (51)	
251–500	35 (21)	1 (2)	2 (6)	38 (14)	
>500	3 (2)	59 (95)	29 (94)	91 (35)	
**Length of Commute**					<0.001 ^a^
0–10 min	114 (67)	12 (19)	3 (10)	129 (50)	
11–30 min	47 (28)	30 (48)	11 (35)	88 (34)	
>30 min	6 (4)	20 (32)	17 (55)	43 (16)	
**Geographic Proximity to work**					<0.001 ^a^
Yes	153 (91)	28 (45)	5 (16)	186 (71)	
No	15 (9)	34 (55)	26 (84)	75 (29)	

^a^: indicates significant effects when *p* < 0.05; CHAs: Community Health Agents; *Pardo*: refers to mixed races, such as mulattos [49].

**Table 3:** Standardized estimated direct effects from fully-adjusted structural equation model.

	Service Integration	
	B (SE)	*p*-Value
**Demographic**		
CHAs vs. physicians	0.373	0.118
Nurses vs. physicians	0.043	0.814
Race: black vs. *pardo*	0.012	0.92
Race: white vs. *pardo*	−0.182	0.125
Male vs. Female	−0.19	0.128
Age	0.15	0.236
**Job Context**		
FHS experience	0.258 ^b^	0.06
Caseload	0.05	0.736
Commute 0–10 min vs. >30 min	−0.122	0.595
Commute 11–30 min vs. >30 min	0.219	0.237
Geographic proximity to work	−0.085	0.629
**Individual factors**		
Confidence	0.322 ^a^	0.02
Knowledge & skills	0.448 ^a^	0.006
Familarity with community	0.153	0.177
Perseverance	0.237 ^b^	0.036
Efficacy of FHS teams	−0.073	0.536
**Job Characteristics**		
Interprofessional collaboration	−0.209	0.121
Consumer-Input	0.033	0.819
Skill variety	0.355 ^a^	0.017
Work-methods autonomy	−0.222 ^b^	0.097
Decision-making autonomy	−0.237 ^b^	0.075
**Organizational factors**		
Work resources	–0.063	0.677
Work conditions	0.044	0.756

^a^: indicates significant effects when *p* < 0.05; ^b^: indicates significant effects when *p* < 0.10; B: Path Coefficients.
